# Chemotion-ELN part 2: adaption of an embedded Ketcher editor to advanced research applications

**DOI:** 10.1186/s13321-018-0292-9

**Published:** 2018-08-13

**Authors:** Serhii Kotov, Pierre Tremouilhac, Nicole Jung, Stefan Bräse

**Affiliations:** 1 Institute of Toxicology and Genetics, Karlsruhe Institute of Technology, Hermann-von-Helmholtz-Platz 1, 76344 Eggenstein-Leopoldshafen, Germany; 2 Institute of Organic Chemistry, Karlsruhe Institute of Technology, Fritz-Haber-Weg 6, 76131 Karlsruhe, Germany; 3 Present Address: Nürnberg, Germany

**Keywords:** Molecule editor, Electronic Lab Journal, Open Source software, Ruby on Rails, JavaScript, Solid supports

## Abstract

The Ketcher editor, available as an Open Source software package for drawing chemical structures, has been expanded to include several features that allow storage, management and application of templates, as well as the use of symbols for a planning and processing of solid phase synthesis. In addition, tools for the drawing of coordinative bonds to represent e.g. organometallic compounds were added. The editor has been implemented into an Electronic Lab Notebook (ELN) application which enables the use of the Ketcher editor for advanced operations in chemistry research. The developments of the ELN-integrated Ketcher (ketcher-rails) support the retrieval of identifiers and structure-related information from external databases and the molecule-based calculation of analytical values. The reworked editor can be used to generate molecular structures in reaction templates and to generate syntheses plans.

**Electronic supplementary material:**

The online version of this article (10.1186/s13321-018-0292-9) contains supplementary material, which is available to authorized users.

## Background

The definition, storage and search of chemical information in electronic laboratory notebooks (ELNs), databases or repositories depends on the availability of chemical editors to draw the molecular structures. Even the creation of chemical names and unique identifiers, as a prerequisite for the correlation of chemical data with descriptions or results, relies on the availability of electronic systems to input structural information [1, 2]. Chemical structure or molecule editors are necessary for the processing of chemical structures in almost every field of chemical research, particularly in organic, inorganic and polymer chemistry, and chemical biology. They are used as standalone software for drawing molecular structures in scientific reports and manuscripts for the publication of research data. Moreover, they are also used as integrated systems [3] for fast and error-free processing of chemical information in diverse software applications [4]. The importance of ELN-integrated editors and the use of digital information management and storage over the whole research process is constantly growing [1, 5, 6]. Especially the storage of reaction data in machine readable formats (available via a processing of the drawings in molecule editors) has high impact as it can be used for the in silico calculation of new reaction rules and the formation of reaction databases enriched with research data [7–9]. In addition, molecule editors are very important to visualize chemical structures to improve their interpretation and understanding [10–13]. Several chemical structure editors have been developed in the past and are used in academia and industry. ChemDraw developed by CambridgeSoft (now a product of PerkinElmer) [14] and ISISDraw developed by MDL Information Systems (now a product of BIOVIA), are among the most prominent editors [15]. Other examples of molecule editors are VectorMol (Sciformation) [16], JChemPaint [17], JSME [18], MarvinSketch (ChemAxon) [19], ChemSketch (ACD) [20], ChemDoodle (iChemLabs) [21], and Ketcher (EPAM Life Sciences) [22]. In particular, the commercial editors, such as ChemDraw, offer a wide range of possible features which allow their use in many fields of chemistry research. However due to the unavailability of the source code for commercial software, adaptions of the molecule editor to the user’s needs are not possible, and the embedding and use of the editor in a flexible (ELN) environment is limited. Both arguments constrain the utility of the advanced commercial editors which reveals the need for continuous work to improve Open Source software. The Ketcher editor is one of the few developments that are available as Open Source and offers a strong basis of already implemented features for use in chemical research. Ketcher is a web-based molecule editor, written in JavaScript, providing options for stereochemistry support, standard 2D geometry optimization (clean up), (de)aromatization, a list of templates for cycles or chain functions, atom hotkeys and R-group labeling tools. Ketcher can import and export molfile/rxnfile, as well as SMILES and supports scalable vector graphics (SVG) or vector markup language (VML) for rendering [23]. Evaluating suitable molecule editors that would work on our current research projects, we found Ketcher to be the most comprehensive, most flexible and best documented Open Source software and decided to use it as a basis for new developments. Additionally, the Ketcher editor can be used on tablets and smartphones which allows more flexibility with diverse devices. The Ketcher editor is well established in the chemistry community and is offered for search functions by e.g. Reaxys or ChemSpider [24]. It is also still under (Open Source) development by the company EPAM [25] which will probably allow a merge of the achievements of our development team with those of EPAM in future. In this manuscript, our efforts towards the improvement of Ketcher editor as a modern and flexible Open Source 2D molecule editor are described. This work has resulted in an advanced molecule editor designed to fulfill the requirements of current chemical research with the addition of several new editing tools, while also offering new features through its interaction in the environment of an ELN. A prerequisite for such a beneficial symbiosis is therefore the implementation of the molecule editor into a flexible and powerful ELN, which was successfully shown at the beginning of this project by embedding ketcher-rails into the Chemotion-ELN, a development of our research group which has been reported earlier [26].

## Implementation

**Fig. 1:**
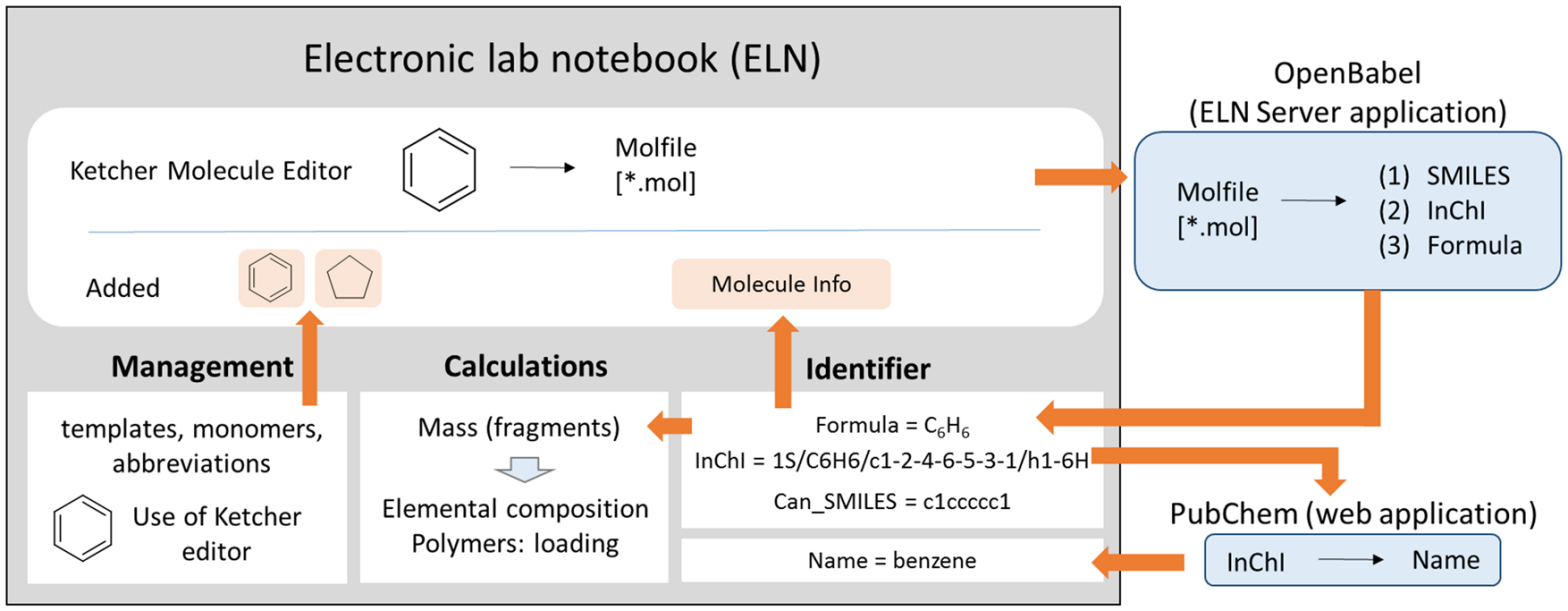
The implementation of Ketcher with Chemotion-ELN using ketcher-rails

The herein described developments base on the last stable, well documented Open Source code of Ketcher (Version 1.1-beta, formerly from GGA-software). A newer version of Ketcher (Ketcher 2.0) is currently under development by the company EPAM [27] and will offer several improvements in comparison to the original software, including the generation of user defined templates and calculated information on the given molecules. We used the established Ketcher 1.1 as Source Code, because Ketcher 2.0 is still in an alpha version (information taken from the provider EPAM) but we intend to upgrade our code to Ketcher 2.0 as soon as it is available as a stable version. Our implementation of Ketcher and its integration into an ELN required a significant optimization and rebuild as a Ruby software package (gem) called ketcher-rails. This allows easier embedding of Ketcher to any Rails application and also enables the use of numerous features of the Ruby on Rails framework. Many of our developed functions benefit from the interaction of the ketcher-rails editor with chemotion-ELN which was programmed in Ruby, Javascript, HTML, and CSS. The backend server is built using the Ruby on Rails framework with PostgreSQL relational database, while the front-end user interface is mainly constructed with the ReactJS framework to serve a single page application. Embedding Ketcher into the Ruby software package allows it to benefit from the flexibility of the ruby programming language. Additionally, embedding it into a web service that uses Ruby on Rails allows for the use of more features to improve the performance of the molecule editor significantly. The embedded ketcher-rails uses the so-called Rails assets pipeline feature that can compile all JavaScript and CSS files into one file and store it on the user’s computer until any of the files are changed. This prevents unnecessary duplicate data transfer, and significantly improves performance, especially in network connections with low throughput and high latency. The same procedure is applied to image files that can be generated and changed by the user, including the regeneration of the compiled image. The technology used for images is called “CSS sprites”. It is used to combine several images into one image and to render specific partitions by CSS language. Therefore, multiple network requests for each image are reduced to a single request for a large image. The molecule editor is delivered as a Ruby software package which is easily integrated to any Ruby-on-Rails web application. Ketcher-rails is a rails ‘engine’; it brings its own MVC components and DB tables into the main rails application. The main function of an embedded molecule editor is the drawing of chemical structures which are registered as molecules and can be used for any chemical application managed by an ELN. For example, typical user cases include the creation of a chemical structure and its storage in the chemical database for commercial compounds, the use of a chemical structure to describe a starting material, reagent or product of a chemical reaction or the definition of a chemical structure as a sample placed for a biological investigation. The procedure taking place in the background during the registration of a chemical structure as a molecule includes several steps. First, a molfile (.*mol) as a standard exchange format is created and transferred to OpenBabel. The use of OpenBabel allows the retrieval of molecule identifiers like SMILES, InChI, and InChIkey. The information on the InChIkey is then used for a request at PubChem for the availability of the chemical name of the given molecule (Fig. 1). The requested information from external sources is stored and visible in the different ELN data fields or used for functions within the ELN e.g. the template generation and management of diverse molecule based calculations. A complete summary of the information that is retrieved, used or stored from third-party services is listed in the Additional file 1: Table S1.

## Results

### Information and identifiers

The obtained identifiers and names of molecules are not only used for the completion of molecule forms of the ELN, but are also directly available in the UI of the molecule editor. This direct availability of information is of particular interest, as structures and sub-structures can be investigated in a very fast manner e.g. according to their molecular weight and exact mass without the need to save the information in the ELN (please see Additional file 1: Figure S1).

### Drawing and processing of coordinative bonds

**Fig. 2:**
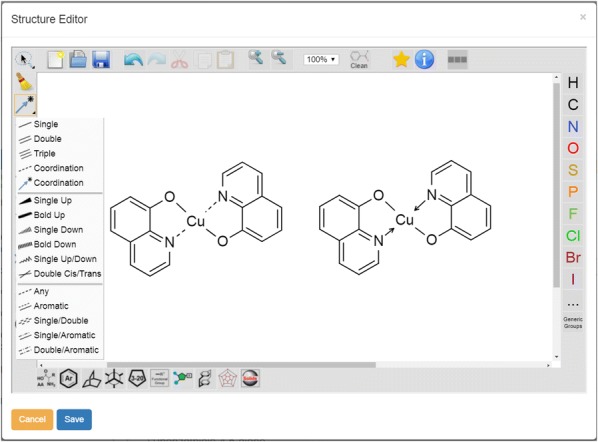
Coordinative bonds as a new function supported by the ketcher-rails editor. The function can be used in form of dashed bonds or donating bonds

The availability of coordinative bonds has been a highly demanded feature by the students using the Chemotion ELN. We therefore implemented a basic functionality that allows to draw and store e.g. organometallic structures (Fig. 2). The molfile format V3000 officially supports, since November 2011, coordinative bonds through a type 9 declaration in the bond block. Ketcher reads molecule structures in V3000 molfile, but exports in V2000 format. However, the type 9 declaration could be backported with V2000 format. The coordinative bonds can be drawn by selection of either a dashed bond or a dative bond symbol in the ketcher-rails UI.

### Management of common templates and template categories

The ELN-embedded ketcher-rails editor was modified to facilitate the input of structures and to maximize its benefits for the user. While the original Ketcher editor offers only a small selection of standard templates by default, a general approach towards the addition of common templates that are persisted in the Chemotion-ELN DB was developed. To this aim, the ELN serves as an environment for the ketcher-rails editor to allow the management of templates through an advanced management model. Within this model, a moderator role was implemented which restricts the permissions of the template registration only to authorized users. The authorized moderator has access to a special UI of the ELN which allows the management of (1) common template categories, (2) single templates, (3) abbreviations, and (4) monomers for the generation of oligomeric structures. Only a moderator can create, edit or delete template categories or common templates (see Additional file 1: Figure S2). The currently available template categories supported by ketcher-rails v0.1.3 and the amount of currently assigned templates are shown in Table 1.

**Table 1:**
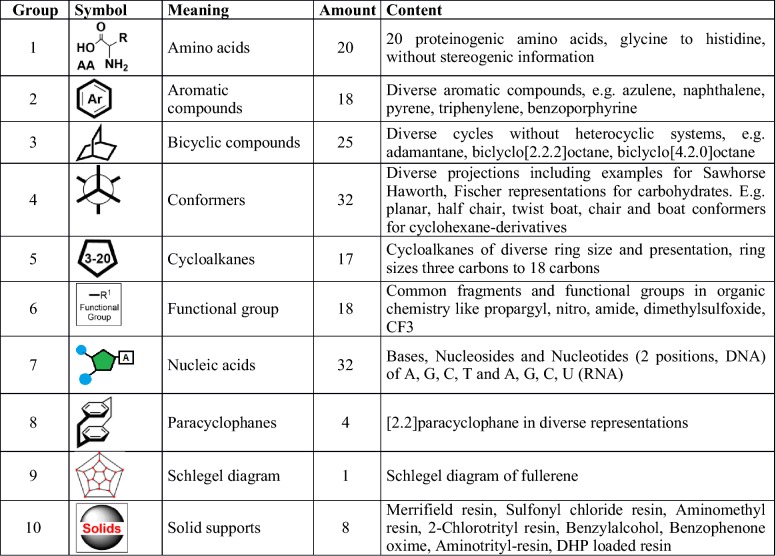
Main template groups, their symbols and single members

The creation of new templates and the assignment to a template category is managed by an input UI that supports the drawing of new templates via the ketcher-rails editor, the use of a molfile for the import of one molecule or the use of a sd file (sdf) which contains multiple chemical table files. The imported structures are saved and can be edited at any time to add the name of the template, the category to which it belongs, and to define the status of the template generation. While all loaded structures obtain the status “pending” automatically, only templates of which the status is changed into “approved” are passed to the molecule editor’s common template selection. Once the template is added to the template list with an approved status, the user can select the template via choosing the corresponding template category. A list of all category members is shown and the desired template can be added to the ketcher-rails editor UI (see Additional file 1: Figure S3). Templates which are not in the desired presentation can be improved via the ketcher-rails clean up function (see Additional file 1: Figure S4). The data for several templates and template categories are available with the current ketcher-rails and can be imported through a rails rake task to populate the ketcher-rails DB tables. This allows the ELN users to benefit from our previously imported structures. The ten currently available template groups, which are represented with an icon are summarized in Table 1.

### Custom templates

**Fig. 3:**
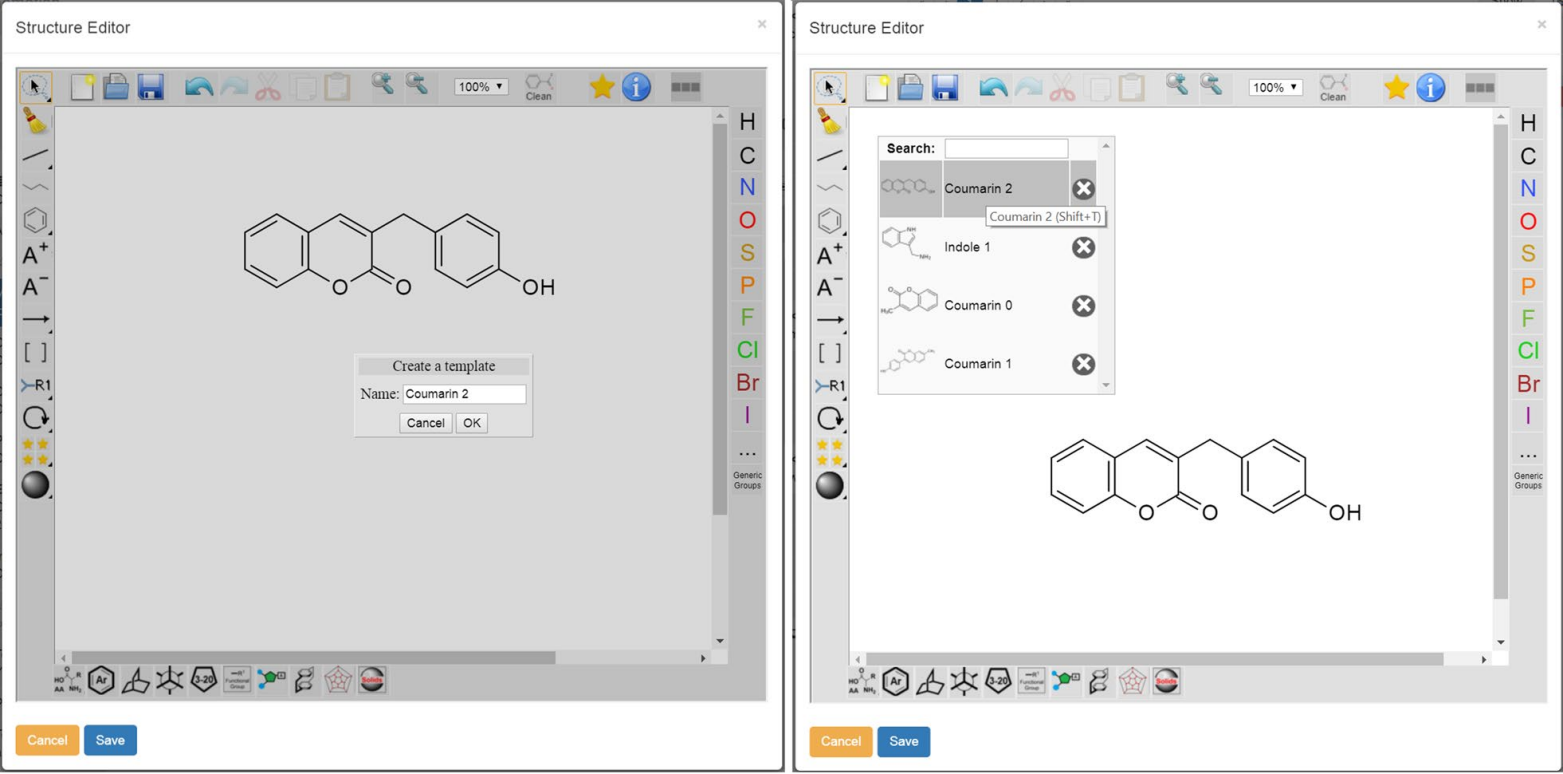
UI for the creation (left) and storage (right) of user-defined templates

Regarding the ketcher-rails editor embedded in our ELN, the most often requested feature by users was the availability of additional user defined templates, in addition to the above mentioned standard templates. The creation of the user-defined templates enables a fast input and change of chemical structures combined with a basic management of the stored structures for the assignment of a name, the text-based search for saved structures and their deletion. The user can either draw a molecule from scratch, can import a molfile to the editor, or open and modify an existing molecule from the ELN to load the necessary structural information (Fig. 3a). The given structure is added automatically to the user’s list of templates with the specified name (Fig. 3b) and the corresponding image of the template is generated as a SVG and a smaller PNG image to be used as an icon. The feature is designed to be used as easy-to-access collection of the most used chemical structure templates. Each template created by a user is restricted to be accessed only by the creator.

### Definition of oligomers and input to the editor

As organic chemistry is not only the chemistry of small molecules but also covers the synthesis and use of oligomeric to polymeric material, a suitable molecule editor should reflect mechanisms for a fast input of structures with diverse repetitions of known components. The definition of these components should also be adaptable to the user group of the editor or the ELN to which it is embedded. Therefore, a registration process was designed based on the above described template registration. Several additional requirements had to be implemented because of the more complex application of these types of templates. One of the most important requirements for the registration of monomers is the definition of attachment points which have to be assigned to particular atoms of the monomer. This was solved via the information panel already available in the ketcher-rails editor. The panel was complemented with an additional checkbox that allows the assignment of not only one atom to an attachment point but also the assignment of a second atom to a secondary attachment point (see Additional file 1: Figure S5). We designed the oligomer-generation tool for peptoid-drawings as this function was requested very often in our research group but the structure of the oligomer-building function allows also the creation other types of oligomers since the monomer generation and the assignment of attachment points can be adapted to the user’s preferences. In a previous step, several monomers with common abbreviations according to peptoid-monomer nomenclature were added as monomer templates. The desired, exemplarily chosen sequence (N2Ph-N1cPr-N2iPr-N2Me-N3m-N1ph-N1ppg) was added to the editor giving the desired product as output view in the ketcher-rails editor. Several adaptions can be made to improve the representation of the given peptoid. The user can, for example, switch from normal to reversed order which changes the sequence order from N to C terminus and vice versa (see Additional file 1: Figure S6).

### Polymer supported reactions and reactions on surfaces

**Fig. 4:**
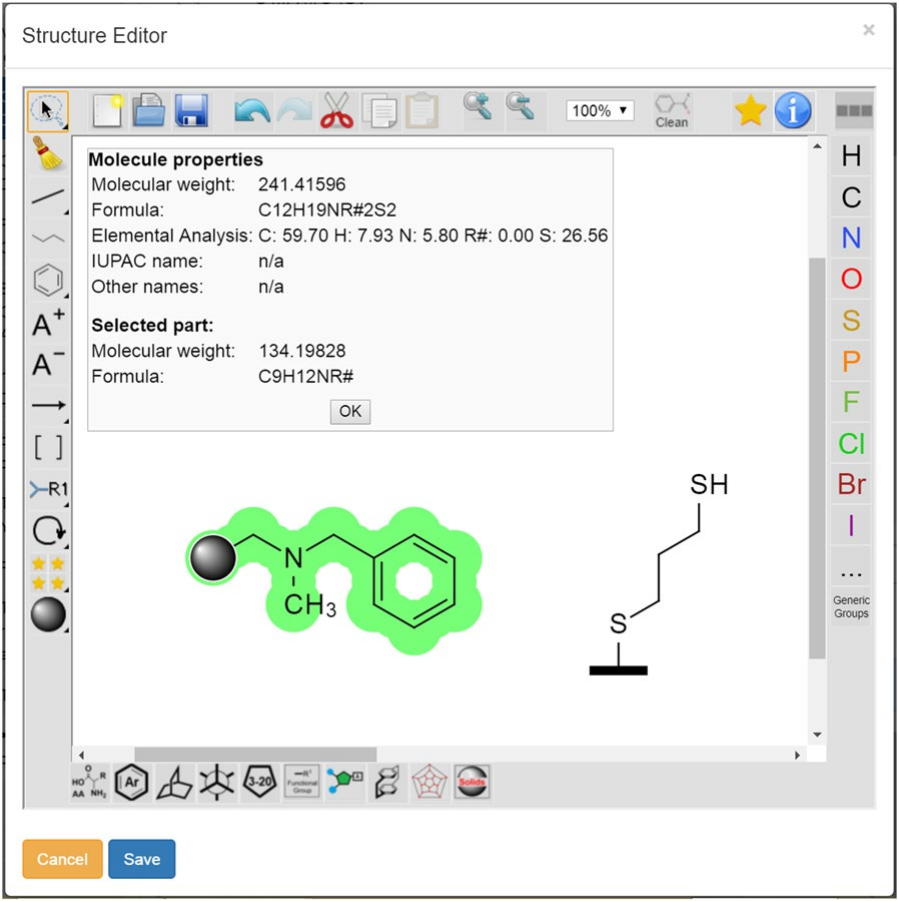
Input and presentation of solid supported and surface-bound structures. Visibility of additional information for the defined part in the information field

The work with an embedded editor requiring the definition of well-defined structures involves problems concerning the registration and processing of polymers which are at least to some extent undefined. To the best of our knowledge, challenges to process polymers as partially undefined structures have not been solved and current editors do not tackle this problem. The latter issue still causes severe limitations for the use of electronic management and storage systems in the field of chemistry. In order to allow the presentation and processing of molecules immobilized on polymers, we introduce a polymer symbol and a symbol for surfaces to be used in combination with the ELN environment to process information for adequate calculations with the undefined molecule. This solution can be applied to all solid-supported reactions as it reflects the constitution of the polymer (elemental composition) which is necessary for reaction planning and calculation. However, for advanced applications in polymer chemistry, the herein suggested model has to be further improved and extended. According to current developments, solid-supported molecules are created by adding the commonly used symbol for polymers to a chemical structure (Fig. 4).

**Fig. 5:**
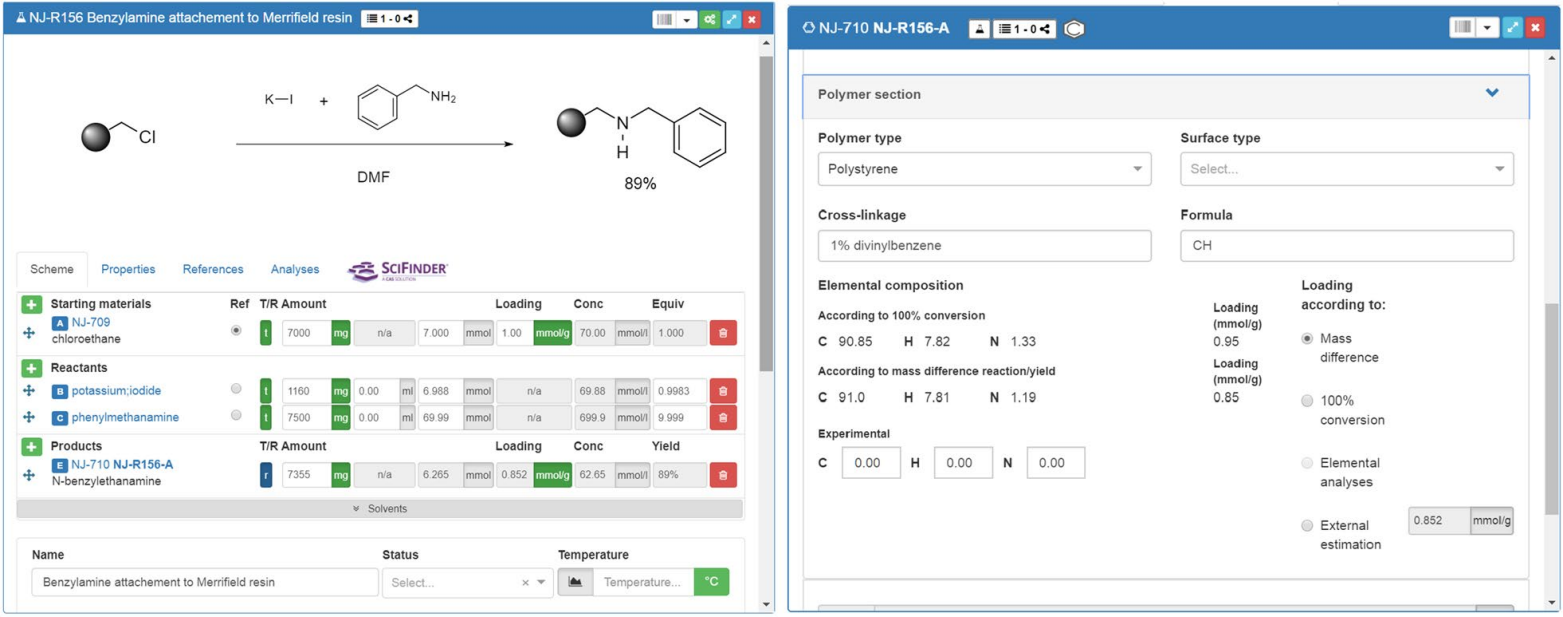
Reaction planning and documentation with the ketcher-rails editor allowing to register polymer supported molecules within an ELN environment. Left: Polymer reaction table including loading. Right: Calculations with polymer supported material. Diverse possible methods to calculate the outcome of polymer supported reactions and expected elemental analysis data

The polymer-connected structure is then passed to a molfile by substitution of the polymer symbol with a methyl group as a chemically similar replacement. The detailed definition of the polymeric part of the molecule (polymer type, cross linkage, average formula of polymer composition, and loading etc.) is done in the ELN-UI where the user can add information on type and composition of the polymer. The latter additional data are used for the calculation of sample-specific values like the elemental composition of the polymeric material. Separating the classification of the molecule via the molecule editor and the definition of the polymeric material in the ELN environment results in the availability of two datasets: a molfile of an approximated reactive molecule site and the information about the combined composition of the polymeric material. The molfile allows the query of information for the defined part of the molecule via OpenBabel and PubChem while being searchable in chemistry databases. The information on the polymeric material can be used for the differentiation of different types of polymers for e.g. loading-dependent calculations. One example for such a calculation is given in Fig. 5 and further details and examples can be retrieved from the supporting information (Sect. 4). The ELN currently supports four types of calculations for polymers with known [calculation (1)] and unknown compositions [calculations (2)–(4)].

1. Calculation of the elemental composition for a given polymer type with information about loading of the material, its formula, and the formula of the immobilized non-polymeric part.
2. The calculation of the yield of the polymer-supported reaction is obtained based on the amount and properties of the starting material (mass and loading) and the obtained mass of the polymer-supported reaction product.
3. The ELN supports further calculations for the yield of the polymer-supported reaction, based on the amount and properties of the starting material and the obtained elemental analysis of the polymer-supported reaction product.
4. The calculation of the loading of the polymer-supported material can be done for an assumed full conversion based on the obtained amount and properties of the starting material, and the obtained mass of the polymer-supported reaction product.

## User feedback and reflections

Technically, the new ketcher rails-editor covers the main features that are also available by the well-known commercial ChemDraw editor. Several features are still missing but other actions can be managed additionally, therefore the establishment of the editor as a standard tool was considered to be relatively easy. We became aware that the acceptance of the ketcher-rails editor strongly depends on the age and experience of the scientists. Users who are long-term users of ChemDraw disliked the switch to an alternative software and were constantly asking for the embedding of ChemDraw to the Chemotion-ELN even though the necessary functions were available by the use of ketcher-rails as well. This adaption process takes some time for long-term users of ChemDraw but the available benefits through the ELN integration accelerate the acceptance in general. The most requested features to be added to the original Ketcher editor were the creation of user defined templates and the option to draw coordinative bonds. The latter feature was needed by many users working in the field of catalysis and the integration of the coordinative bond type convinced additional interested people to use ketcher-rails. One point to be mentioned which leads to inconveniences for some scientists is the fact that ketcher-rails is developed as a professional tool allowing the processing of structures for their addition to databases which includes no tolerance of wrong structures. The latter fact is very advantageous to guide users to a well-written documentation but is not favorable for users that tend to a more “quick and dirty” documentation. In particular through the connection to the ELN, non-accurate drawings have direct influence on the availability of the correct name of the compound and its calculated properties and values.

## Conclusion

The molecule editor Ketcher was extended concerning its functionality and was embedded to an ELN environment which enables the use for advanced applications in chemistry research. The developments on the ELN-integrated ketcher-rails editor support the retrieval of identifiers and structure-related information from external databases, and the molecule-based calculation of analytical values. A model for the addition of common templates and the definition of user defined templates was created to facilitate the input of molecules and the adaption of the editor’s possibilities to the needs of research. The embedded editor offers a straightforward solution for the representation of polymer supported molecules, their processing with PubChem or OpenBabel, and their use for calculations with polymeric material. In addition, the drawing of coordinative bonds was enabled which allows to draw e.g. organometallic complexes. In summary, our additions and improvements to the original Ketcher source code and the interaction with the ELN allow researchers to manage their data more efficiently and will support the efforts of the community to adapt the current chemistry research infrastructure to their needs. We hope that the developments will support the efforts to develop other suitable scientific applications or Open Source software for chemists.

## Additional file

**Additional file 1.** Technical aspects and details of the software and programming e.g. the use of predefined templates and their moderation, the installation requirements and the details of the Docker image, and explanations for the use of ketcher-rails in other rails applications. Some additional explanations concerning the interaction of ELN and ketcher-rails are given, including several images to illustrate selected functions of the editor.

## Abbreviations

ELN: Electronic Laboratory Notebook
MVC: model-view-controller
CSS: Cascading Style Sheets
DB: database
UI: user interface
