# Supplementary material for: Chemotion-ELN part 2: adaption of an embedded Ketcher editor to advanced research applications
Source: J Cheminform. 2018 Aug 13;10:38. doi: 10.1186/s13321-018-0292-9 (PMC6089857; doi:10.1186/s13321-018-0292-9)
Supplement: Supplementary file 1 — Additional file 1. Technical aspects and details of the software and programming e.g. the use of predefined templates and their moderation, the installation requirements and the details of the Docker image, and explanations for the use of ketcher-rails in other rails applications. Some additional explanations concerning the interaction of ELN and ketcher-rails are given, including several images to illustrate selected functions of the editor. [file 13321_2018_292_MOESM1_ESM.docx]

Chemotion-ELN Part 2: Adaption of an embedded Ketcher editor to advanced research applications.

Serhii Kotov^a^, Pierre Tremouilhac^a^, Nicole Jung* ^a,b^, Stefan Bräse* ^a,b^

^a^Institute of Toxicology and Genetics, Karlsruhe Institute of Technology, Hermann-von-Helmholtz-Platz 1, 76344 Eggenstein-Leopoldshafen, Germany; ^b^Institute of Organic Chemistry, Karlsruhe Institute of Technology, Fritz-Haber-Weg 6, 76131 Karlsruhe, Germany.

Contents

[1. Common templates import and moderation 3](#_Toc518247586)

[1.1 Import of common templates and categories 3](#_Toc518247587)

[1.2 Set a user account as template moderator 3](#_Toc518247588)

[1.3 Availability of the Docker image 3](#_Toc518247589)

[1.4 Installing ketcher-rails into another Rails application 4](#_Toc518247590)

[2. Summary of chemical structure information 6](#_Toc518247591)

[3. Functions of the Ketcher Molecule editor in different versions 6](#_Toc518247592)

[4. Implemented calculations for solid supports 7](#_Toc518247593)

[5. Additional pictures to illustrate the application 9](#_Toc518247594)

# 1. Common templates import and moderation

The ELN can be tested at <https://eln.chemotion.net>. The implementation of the ketcher editor can also be accessed directly at <https://eln.chemotion.net/ketcher/demo> .

##

## 1.1 Import of common templates and categories

Data seeds to populate the ketcher-rails ‘common templates’ and ‘template categories’ tables are available and can be loaded into the current db using rails rake tasks by the server administrator (note that the DB schema should be up to date):

- with the task ‘db:seed’: in the rails main application db/seed.rb file, add the following line: *Ketcherails::Engine.load_seed*.

Thereafter run *bundle exec rake db:seed*

- Or with the task ‘ketcherails:import:common_templates’:

run *bundle exec rake ketcherails:import:common_templates*

Warning: this will overwrite previously created common templates and categories.

## 1.2 Set a user account as template moderator

The server administrator can set a specific user account as template moderator to give the users management rights over the templates. Start the rails console and run:

*User.find_by(email: ‘user.email@eln.edu’)&.update!(is_templates_moderator: true)*

where user.email@eln.edu is the email of the user to be made template moderator.

## 1.3 Availability of the Docker image

A Docker image for the Chemotion_ELN is available at https://hub.docker.com/r/complat/chemotion_eln (see the installation guide INSTALL.md^[[1]](#footnote-1)^). This provides the default common templates and a generic user account for template moderation (template.moderator@eln.edu, pw: @eln.edu).

In addition, a VM image can be retrieved from:

https://git.scc.kit.edu/ComPlat/chemotion_eln_server/wikis/vm-template

## 1.4 Installing ketcher-rails into another Rails application

Although not mandatory, the Rails application implementing the ketcher-rails should have a user model for Warden-based authentication (<https://github.com/wardencommunity/warden/wiki>).

The README notes on <https://github.com/ComPlat/ketcher-rails> gives detailed information and the source of a demo app showing how to implement is available at <https://github.com/ComPlat/ketcher-rails-test_app>

The information is given following:

## Ketcherails

### Description

This gem provides the possibility to use Ketcher v1. editor with Rails and contains

server actions implementations.

Ketcher editor has been originally built by GGA Software(https://github.com/ggasoftware/ketcher)

### Usage

This is a self-mountable Rails engine.

Add it to your app Gemfile

*gem 'ketcherails', git: 'https://github.com/ComPlat/ketcher-rails'*

Inkscape should also be installed (`sudo apt-get install inkscape` for Linux).

To take advantage of the molecule common template features, the app should have a user model for Warden based authentication.

The User model should have/expose a boolean attribute 'is_templates_moderator' to authorize the template editing.

Or bypass this by stubbing the current_user (see https://github.com/ComPlat/ketcher-rails-test_app/commit/f9d14e5fd6e8925e1e484f667eaaef998c06a125 blank test app).

The user custom template feature also relies on user Warden authentication.

You need to set the application active_job handler (eg with DelayedJob see https://github.com/ComPlat/ketcher-rails-test_app/commit/f147743e8ada73ebb28c6ce6356b982fde968abc)

Routes are mounted to /ketcher:

- full page editor: /ketcher

- client api demo editor: /ketcher/demo

- Template management pages: /ketcher/common_templates

Insert the ketcher into an iframe; Molfile setter/getter, and SVG getter functions are available :

*<iframe width="80%" height="800" id="ifKetcher" src="/ketcher"></iframe>*

*<script>*

*function ketcher() {*

*const ketcherFrame = document.getElementById('ifKetcher');*

*if (ketcherFrame && ('contentDocument' in ketcherFrame)) {*

*return ketcherFrame.contentWindow.ketcher;*

*}*

*return document.frames['ifKetcher'].window.ketcher;*

*};*

*function getSVG() {*

*document.getElementById("result").innerHTML = ketcher().getSVG();*

*};*

*function getMolfile() {*

*document.getElementById("result").innerHTML = ketcher().getMolfile();*

*};*

*function setMolfile() {*

*const molfile = "\n Ketcher 06271817312D 1 1.00000 0.00000 0\n\n 3 3 0 0 0 999 V2000\n 1.0000 0.0000 0.0000 C 0 0 0 0 0 0 0 0 0 0 0 0\n 0.5000 -0.8660 0.0000 C 0 0 0 0 0 0 0 0 0 0 0 0\n 0.0000 0.0000 0.0000 C 0 0 0 0 0 0 0 0 0 0 0 0\n 1 2 1 0 0 0\n 2 3 1 0 0 0\n 1 3 1 0 0 0\nM END\n$$$$\n"*

*ketcher().setMolecule(molfile);*

*};*

*</script>*

*<button onclick="getSVG()">get SVG</button>*

*<button onclick="getMolfile()">get Molfile</button>*

*<button onclick="setMolfile()">Draw cyclopropane</button>*

(see app/view/ketcherails/ketcher/demo.html, or app/assets/javascripts/ketcher/demo.html, or go to

ketcher/demo in your application)

### License

This project uses GPLv3 license.

# 2. Summary of chemical structure information

**Table S1.** Chemical structure information retrieved from third-party services.

| **Field^a^** | **API^b,c^** | **Description** | **Example** | **Saved^d^** | **Display^e^** |
| --- | --- | --- | --- | --- | --- |
| Formula | OB | Molecule formula | C6H6 | x | x |
| SMILES | OB | SMILES format desc. | c1ccccc1 | x | - |
| Can. SMILES | OB | Canonical SMILES | c1ccccc1 | x | x |
| InchI | OB | InChI-String | InChI=1S/C6H6/c1-2-4-6-5-3-1/h1-6H | x | x |
| InchIkey | OB | InChI-Key | UHOVQNZJYSORNB-UHFFFAOYSA-N | x | - |
| Charge | OB | Total charge | 1 (for +1) | - | - |
| Mol. Wt. | OB | Molecular weight | 78.111840 | x | x |
| Mass | OB | Exact molecular weight | 78.046950 | x | x |
| Spin | OB | Spin multiplicity | 1 | - | - |
| SVG | OB | SVG molecule image | SVG Image | x | (x) |
| FP | OB | Molecule fingerprint | Array [0,2..] | x | - |
| CID | PC | Compound identifier | CID: 241 | x | - |
| IUPAC name | PC | IUPAC name | benzene | x | x |
| Log P | PC | LogP (using XLogP) | 2.1 | - | - |
| Atoms | PC | Parsed atom array | Array of objects | - | - |

^a^Field = comparable to naming of OpenBabel/Pubchem; ^b^OB = OpenBabel; ^c^PC = PubChem^; d^Save = Storage of the information in the database; ^e^Display = use of the information in the ELN/UI.

# 3. Functions of the Ketcher Molecule editor in different versions

**Table S2.** Summary of the functions of the ketcher molecule editor

| Feature | Ketcher  (standalone) | Ketcher  (server) | Ketcher 2.0  (alpha) | Chemotion & ketcher-rails |
| --- | --- | --- | --- | --- |
| Stereochemistry (absolute configuration) | 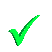 | 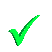 | 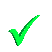 | 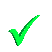 |
| Stereochemistry (relative configuration) | - | - | - | 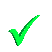 |
| Reactions, reacting centers, atom flags | 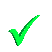 | 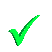 | 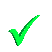 | 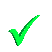 |
| Support of side groups (e.g. generic and superatoms) | 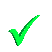 | 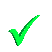 | 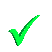 | 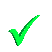 |
| Support of Residues | 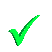 | 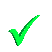 | 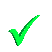 | 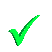 |
| (de)aromatization |  | 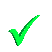 | 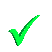 | - |
| Automatic layout (clean up structure) | - | 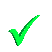 | 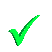 | 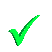 |
| Export [Molfile/Rxnfile](http://lifescience.opensource.epam.com/resources.html#file-formats), as well as SMILES | 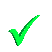 | 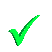 | 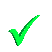 | 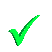* |
| Smiles import | - | 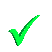 | 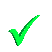 | -* |
| Show molecule information | - | - | 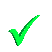 | 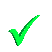 |
| Create and use new template | - | - | 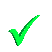 | 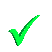 |
| Define new general template group | - | - | - | 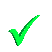 |
| General templates moderation | **-** | **-** | **-** | 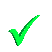 |
| Processing of solid supported material | - | - | - | 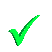 |
| Oligomer input by abbreviations | - | - | - | 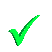 |
| Use as synthesis planning tool | - | - | - | 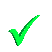 |
| Performance optimizations | - | 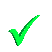 | 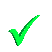 | 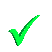 |

* Is possible with Chemotion-ELN and therefore not implemented in ketcher-rails directly.

**Table S3.** Impact of ketcher-rails tools on ELN-functionalities

| Field | Feature | Third Party Tools |
| --- | --- | --- |
| Formatting | (1) Fast addition of novel structures through user-defined templates |  |
|  | (2) Definition and management of new template groups through selected moderators |  |
|  | (3) User defined adaption of molecule style formatting |  |
|  | (4) Automatic molecule layout | OpenBabel |
| Calculations | (5) Calculation of molecular mass, exact mass, composition of elemental analysis (EA) | OpenBabel |
| Information | (6) Retrieval of chemical name | PubChem |
|  | (7) Presence of compounds in PubChem | PubChem |
| Symbols | (8) Representation of solid supports, determination of composition and calculation of related values like EA |  |

# 4. Implemented calculations for solid supports

(1) Calculation of the elemental composition for a given polymer type with information about loading of the material (***L_A_***), its formula (**Form_Pol_**), and the formula of the immobilized non-polymeric part (**Form_IMM_).** The elemental composition is then a result of the combined contents of each element or atom (**EA_atom_**) that is part of the whole polymeric structure (polymer + immobilized molecule, see Figure 4):

(Equ. A) EA_atom_ [%] = 100 * (**A_IMM_** * WR(atom)_IMM_ + **A_POL_** * WR(atom)_POL_)

**Figure 4**. Information, abbreviations and calculations with solid supported molecules for the generation of data for elemental analyses.

If the polymer is a product of a reaction and the composition of the polymer is unknown, the ELN supports the following calculations based on the obtained reaction outcome:

(2) The calculation of the yield of the polymer-supported reaction is obtained based on the amount and properties of the starting material (mass **m_S_**_,_ loading **L_S_**) and the obtained mass of the polymer-supported reaction product (**m_P_**). A prerequisite is the input of information concerning the molecular weight of the immobilized part of starting material **MW(S)_IMM_** and the molecular weight of the immobilized part of product **MW(P)_IMM_** *via* the molecule editor.

(Equ. B)

$$\boldsymbol{yield=}\frac{\frac{\frac{\boldsymbol{1}}{\boldsymbol{L}_{\boldsymbol{A}}}\boldsymbol{*}\boldsymbol{m}_{\boldsymbol{P}}}{\boldsymbol{m}_{\boldsymbol{S}}}\boldsymbol{-1}}{\boldsymbol{MW(S)}_{\mathbf{IMM}}\boldsymbol{-}\boldsymbol{MW(P)}_{\mathbf{IMM}}}$$

The calculations of the reaction outcome allow the direct comparison of the obtained results with the theoretical achievable values. The ELN uses these comparisons in an alarm system, providing information for the ELN users (a) if the experimentally found weight is more than the weight according to a full conversion of the starting material and (b) if the experimental weight is less than the mass of the initially used material. In the few cases where the reaction proceeds with expected loss of molecular weight in the immobilized molecule (MW(P)_IMM_ < MW(S)_IMM_), the ELN warns the user if the experimental weight is less than the expected weight with full conversion.

(3) The ELN supports further the calculation of yield of the polymer-supported reaction based on the amount and properties of the starting material and the obtained elemental analysis of the polymer-supported reaction product.

In this case, the yield is calculated by the difference of the elemental values of product (**EA(P)_atom_**) and starting material (**EA(S)_atom_**) in relation to the expected difference from both values (calculated **EA(P)_atom_ - EA(S)_atom_**) referring to the elements sulfur, nitrogen or carbon as the standard reference element/atom.

(Equ. C)

$$yield=100*\frac{{EA(P)}_{atom}-{EA(S)}_{atom}}{{expected EA(P)}_{atom}-{EA(S)}_{atom}}$$

(4) The calculation of the loading of the polymer-supported material after a reaction on solid supports (**L_P_**) can be calculated for an assumed full conversion based on the obtained amount (**m_S_**) and properties of the starting material and the obtained mass of the polymer-supported reaction product (**m_P_**).

(Equ. D)

$$L_{P}=\frac{L_{S}*m_{S}}{m_{P}}$$

# 5. Additional pictures to illustrate the application

**
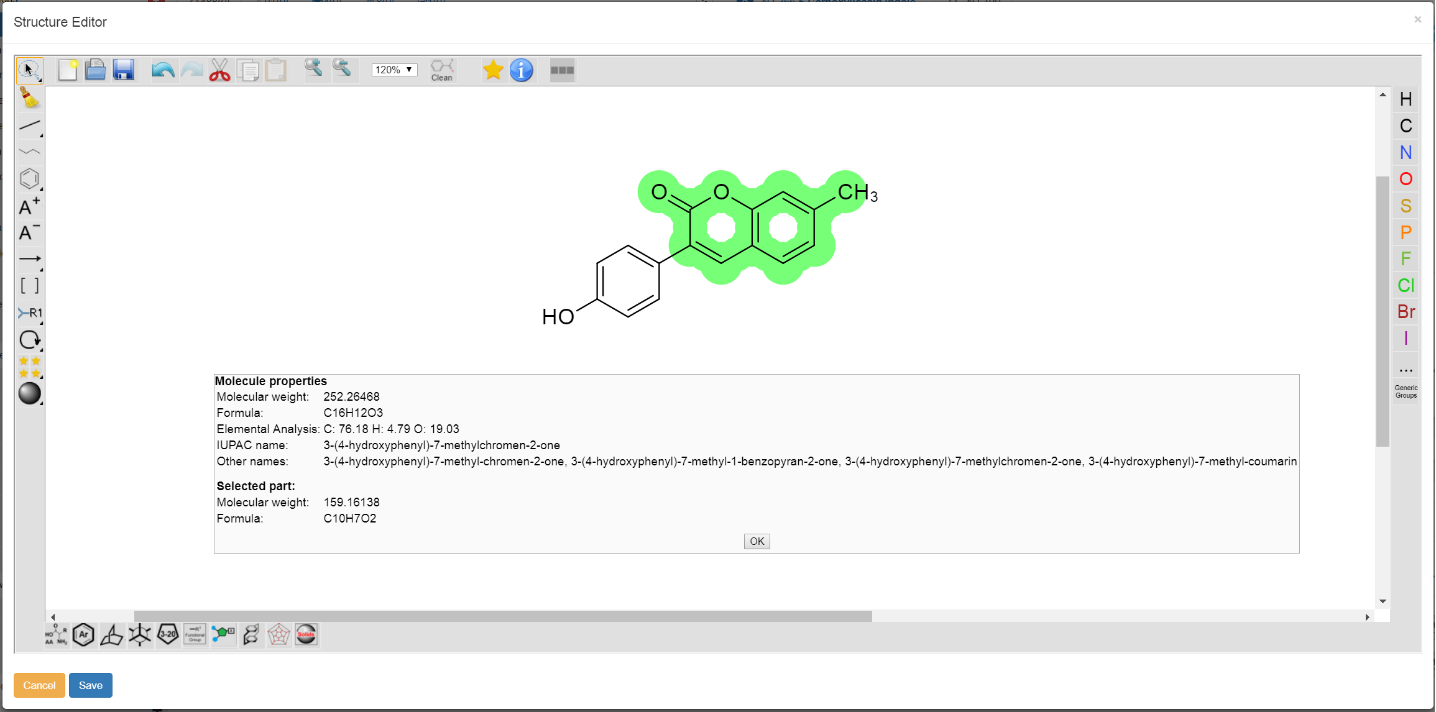
**

**Figure S1.** Availability of structure and substructure mass information in the molecule editor UI.

**
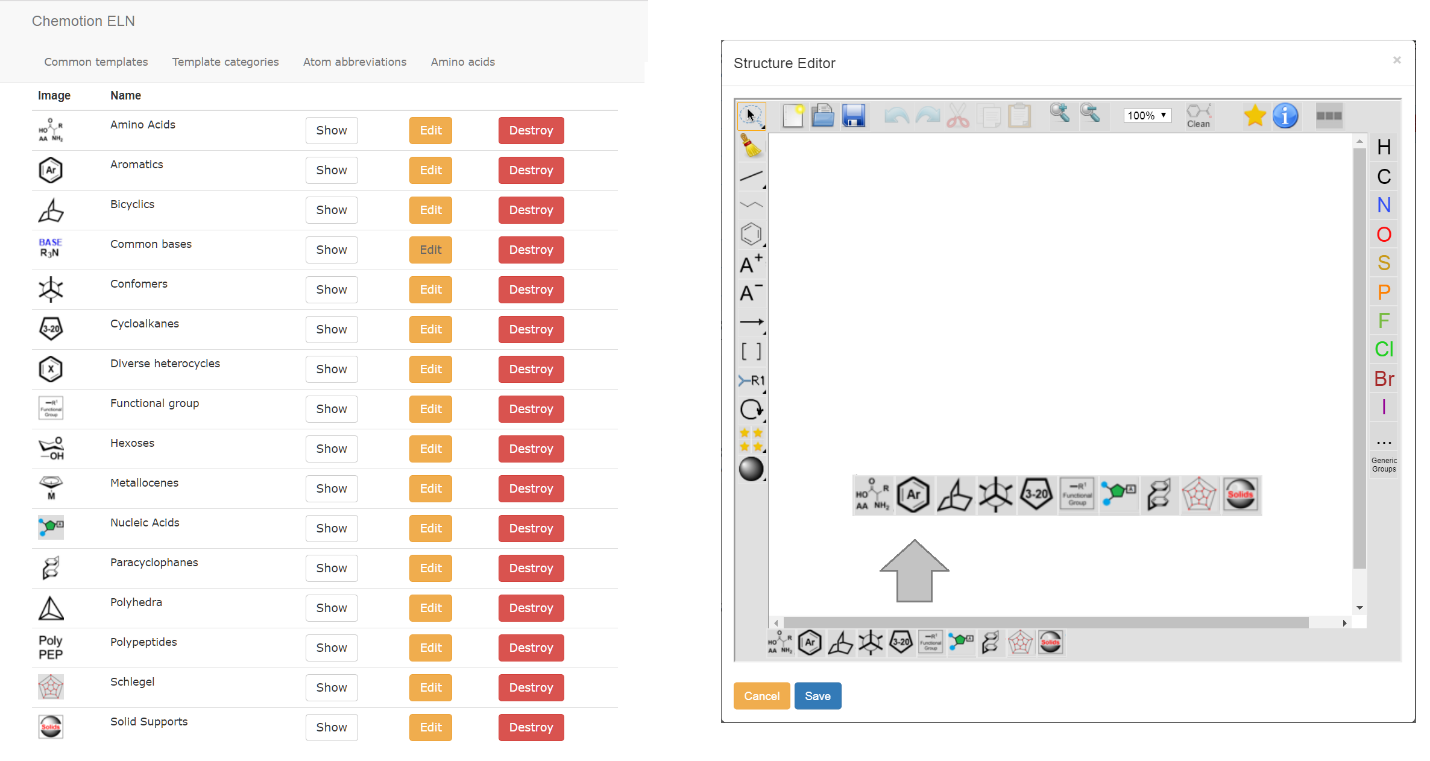
**

**Figure S2.** Workflow for the generation of new template categories via the chemotion-ELN UI and passing to the molecule editor.

**
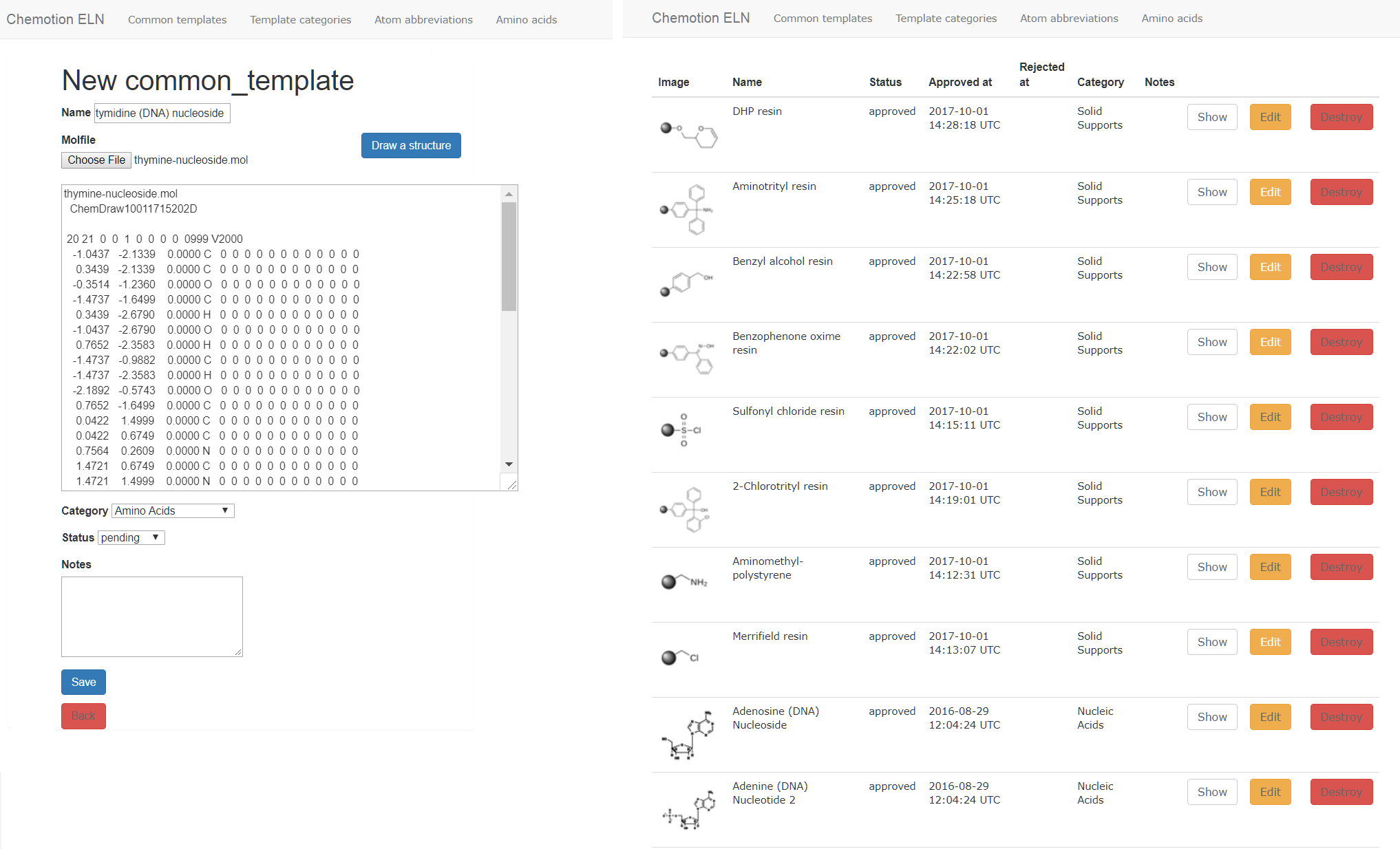
**

**Figure S3.** Workflow for the generation of new templates via the chemotion-ELN UI. Left: Use of structures via upload of molfiles; Right: List of common templates in the management UI of templates.

**
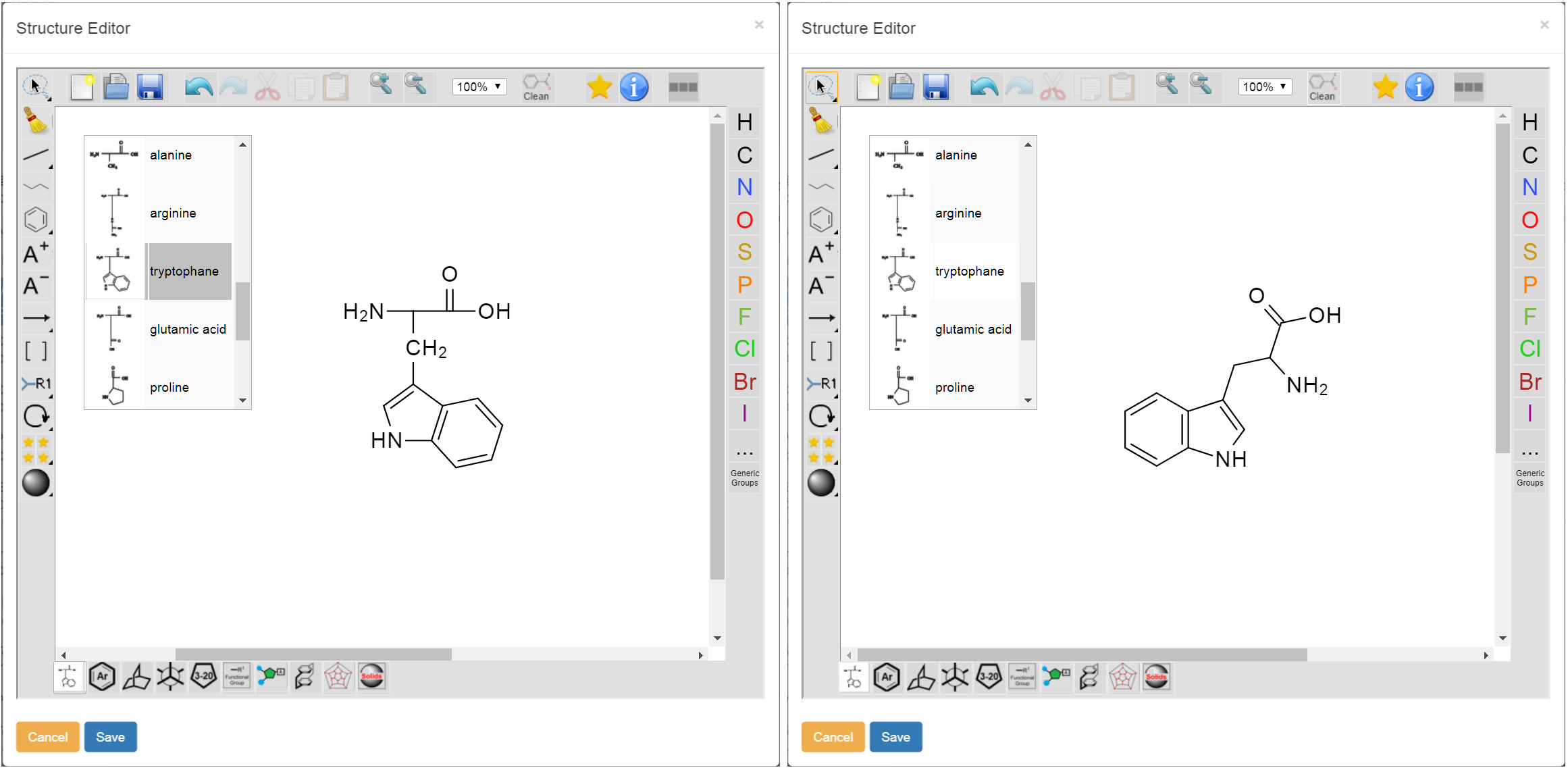
**

**Figure S4.** Using the new templates that were created via the chemotion-ELN UI in the molecule editor. Left: selection of templates from a template category list; Right: structure optimization by using the clean up function.

**
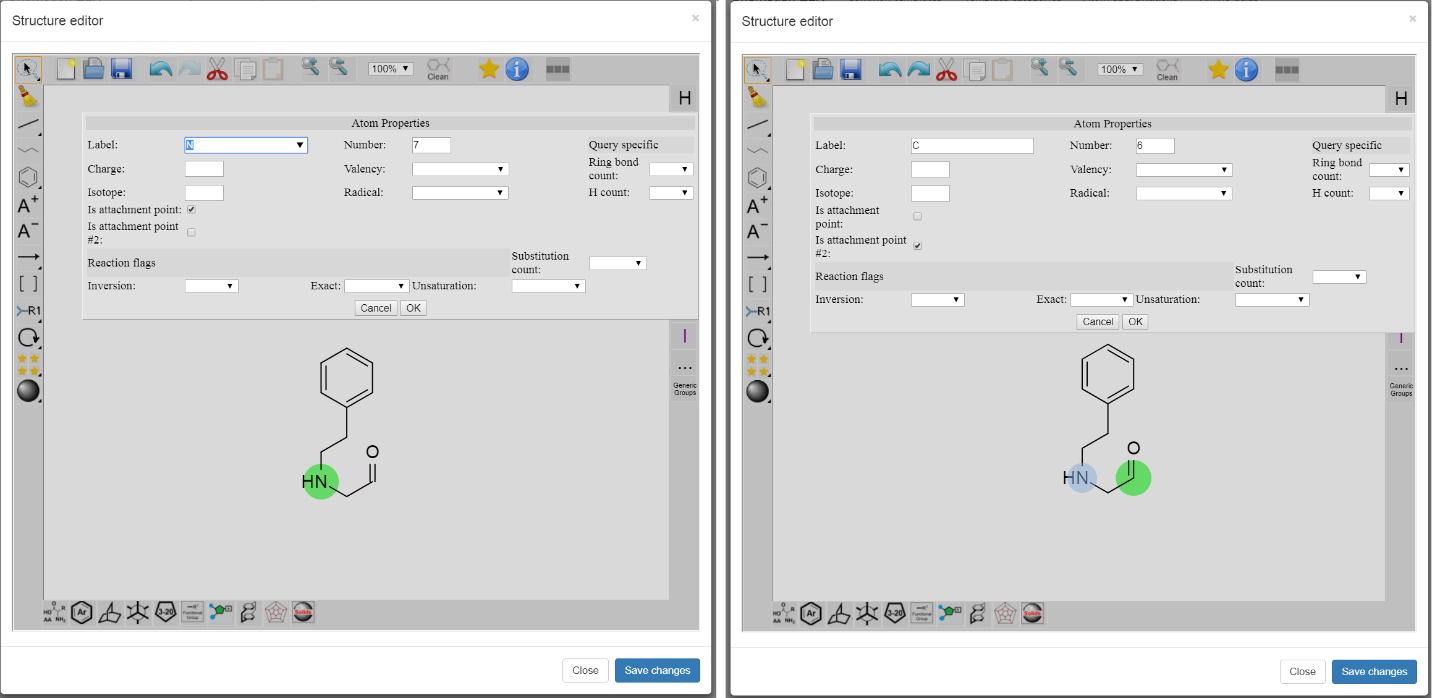
**

**Figure S5.** Procedure to add monomers to the list of available templates. Left: one atom of the monomer has to be assigned to the first attachment point; Right: another atom bears attachment point 2.


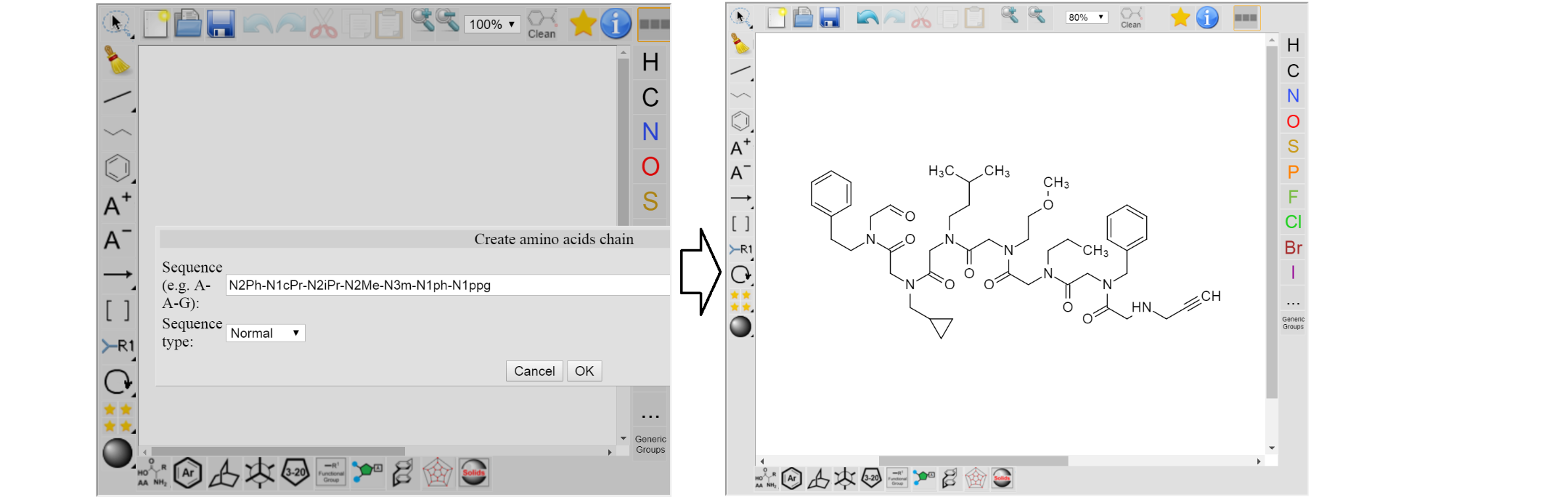


**Figure S6**. Generation of oligomers in the ketcher-rails supported ELN via sequence formation with pre-labeled monomers. Left: Input field for the sequence (sequence given by identifier of monomers); Right: sequence as a result of the transformation.

1. <https://github.com/ComPlat/chemotion_ELN/blob/master/INSTALL.md#docker-setup> [↑](#footnote-ref-1)
